# Modelling local areas of exposure to *Schistosoma japonicum* in a limited survey data environment

**DOI:** 10.1186/s13071-018-3039-6

**Published:** 2018-08-13

**Authors:** Andrea L. Araujo Navas, Ricardo J. Soares Magalhães, Frank Osei, Raffy Jay C. Fornillos, Lydia R. Leonardo, Alfred Stein

**Affiliations:** 1 Faculty of Geo-information Science and Earth Observation (ITC), University of Twente, PO Box 217, 7500 AE Enschede, The Netherlands; 2 UQ Spatial Epidemiology Laboratory, School of Veterinary Science, The University of Queensland, QLD, Gatton, 4343 Australia; 3 Child Health and Environment Program, Child Health Research Centre, The University of Queensland, QLD, South Brisbane, 4101 Australia; 4 Institute of Biology, College of Science, University of the Philippines Diliman, 1101 Quezon, Philippines; 5 Department of Parasitology, College of Public Health, University of the Philippines Manila, 1000 Manila, Philippines

**Keywords:** Schistosomiasis, Spatial modeling, Bayesian network, Exposure uncertainty, Risk factors

## Abstract

**Background:**

Spatial modelling studies of schistosomiasis (SCH) are now commonplace. Covariate values are commonly extracted at survey locations, where infection does not always take place, resulting in an unknown positional exposure mismatch. The present research aims to: (i) describe the nature of the positional exposure mismatch in modelling SCH helminth infections; (ii) delineate exposure areas to correct for such positional mismatch; and (iii) validate exposure areas using human positive cases.

**Methods:**

To delineate exposure areas to *Schistosoma japonicum*, a spatial Bayesian network (sBN) was constructed. It uses data on exposure risk factors such as: potential sites for snails’ accessibility, geographical distribution of snail infection rate, and cost of the community to access nearby water bodies. Prior and conditional probabilities were obtained from the literature and inserted as weights based on their relative contribution to exposure; these probabilities were then used to calculate joint probabilities of exposure within the sBN.

**Results:**

High values of probability of *S. japonicum* exposure correspond to polygons where snails could potentially be present, for instance in wet soils and areas with low slopes, but also where people can easily access water bodies. Low correlation (*R*^2^ = 0.3) was found between the percentage of human cases and the delineated probabilities of exposure when validation buffers are generated over the human cases.

**Conclusions:**

The utility of a probabilistic method for the identification of exposure areas for *S. japonicum*, with wider application for other water-borne infections, was demonstrated. From a public health perspective, the schistosomiasis exposure sBN developed in this study could be used to guide local schistosomiasis control teams to specific potential areas of exposure, and improve efficiency of mass drug administration campaigns in places where people are likely to be exposed to the infection.

**Electronic supplementary material:**

The online version of this article (10.1186/s13071-018-3039-6) contains supplementary material, which is available to authorized users.

## Background

Schistosomiasis (SCH) is a water-borne neglected tropical disease of global public health significance [1, 2]. It affects more than 252 million people worldwide [3], especially human populations living in places where clean water and sanitation are limited [4]*.* Schistosomiasis is known to lead to anaemia, stunted growth and other organ pathologies in school-aged children [5, 6]. Three schistosome species cause the infection*: Schistosoma mansoni*, *S. japonicum* and *S. haematobium. Schistosoma japonicum* is presently endemic in China, Indonesia and the Philippines, and is hard to control due to its zoonotic life-cycle [7]. The life-cycle of *S. japonicum* includes infection of an amphibious snail belonging to several subspecies of *Oncomelania hupensis* as the intermediate host, and humans and other mammalians as definitive hosts [8, 9].

Traditionally, schistosomiasis risk mapping has enabled the identification of at risk populations for targeting mass drug administration campaigns, thus increasing the efficiency of schistosomiasis disease control [10]. Schistosomiasis mapping has been supported by the use of spatial information techniques, such as geographical information systems (GIS), remote sensing and global positioning systems (GPS). Spatial information techniques allow the manipulation of spatially referenced infection data and data on the physical and biological environmental variables [11–14]. Modelling those data in combination allows studying the distribution of communities most at risk schistosomiasis and the role of the geographical variation of environmental exposure factors on schistosomiasis risk [15].

There are a number of errors inherent to spatial information used in geographical epidemiological studies [4]. Most of these errors involve positional measurement errors, where observation and prediction locations are affected by various factors such as GPS inaccuracies, the presence of multiple addresses, geocoding errors, outcome or covariate aggregations, and misalignment between covariates of exposure and disease outcome estimates [15]. The last one is of our current interest and may occur when covariates of exposure are extracted from locations where exposure has not occurred.

Statistical modelling of the spatial distribution of schistosome infections estimates empirical relationships between morbidity indicators (e.g. prevalence or intensity of infection) and risk factors. Risk factors for schistosome exposure include various environmental and socio-economic covariates that help to interpolate the level of infection at unsampled locations [14, 16–18]. Covariates and morbidity indicators are commonly extracted from survey locations such as health centres, hospitals and schools. In most cases, exposure to infection did not occur at survey data locations but at locations where environmental and geographical conditions, together with the level of accessibility to contaminated sites, are optimally exposed. Such exposure locations are usually unknown, resulting in positional mismatch of the surveyed disease values, and the covariates in the model.

To date, methods to account for this type of positional misalignment are scarce. Several studies have used remote sensing data to determine biophysical features of habitats in relation to snail prevalence [19–24], acknowledging that *S. japonicum* transmission is closely related to the distribution of its intermediate host in the environment [9]. Only one study [2] has used these habitats to correct for the positional mismatch when modelling disease infection risk in human populations. Walz et al*.* [2] used high-resolution remote sensing data, environmental field measurements, and ecological data, to model environmental suitability for schistosomiasis-related parasites and snail species. They represented environmental suitability as potential transmission areas that could guide public health interventions to places where people could potentially be infected. Although potential transmission areas were delineated, interactions between humans, hosts, and suitable environments were not taken into account.

These studies suggest that ignoring positional mismatch and its impact on spatial prediction remains largely unquantified in schistosomiasis modelling. Furthermore, the extraction of covariate values in the presence of positional mismatch is a significant source of uncertainty that may influence the efficacy of schistosomiasis control strategies [4]. Therefore, methods to correct for this positional mismatch need to be further investigated [1, 4].

The objective of this study is to develop a schistosomiasis exposure sBN model that maps potential areas of exposure to *S. japonicum*, taking into account human interactions with main sources of infection (i.e. water bodies). To accomplish this objective, we aimed to (i) describe the positional mismatch problem in modelling *S. japonicum* infection; (ii) delineate exposure areas that take into consideration the accessibility cost of people to main sources of infection, and that could be used to correct for this positional mismatch; and to (iii) validate the delineated exposure areas.

## Methods

### Data on human and snail *S. japonicum* infection

In the Philippines *S. japonicum* is endemic in 28 of its 81 provinces [25], with approximately 1.8 million estimated infected people [26]. The disease affects children, adolescents and individuals with high-risk occupations, such as farmers and fishermen [26, 27]. In the Philippines, the smallest administrative division is the barangay, numbering about 22–50 in a municipality.

We used data on human schistosomiasis and snail prevalence of infection, collected in six barangays from Alangalang municipality in Leyte Province in 2015 and 2016. Data were collected by researchers from the College of Public Health and College of Science from the University of the Philippines. Surveyors selected Alangalang municipality because it has the highest prevalence of schistosomiasis (7.5%) from all the 43 municipalities of Leyte Province; within this municipality, they visited the barangays with the highest prevalence of infection from the 54 barangays in Alangalang municipality.

Human positive cases (12 records) were georeferenced at household locations and snails surveys (8 records) were taken from water bodies in close proximity to surveyed households. The recording of all the human case locations (also including negative cases) was not possible due to a lack of manpower and material resources, such as the availability of only one GPS device in the field.

Diagnosis of schistosomiasis in humans was performed using stool examination. Single stool sample was requested per participant with informed consent, coded and prepared following the Kato-Katz method. Each slide prepared was read in the field using a microscope and the presence of *S. japonicum* eggs indicated active infection.

Infection among *O. h. quadrasi* snails was determined by manually crushing the snails in aliquots on a glass slide. Each snail was placed in an aliquot droplet of distilled water, usually three aliquots per glass slide. Snails were gently crushed in between slides and were examined under a conventional stereomicroscope (40×) using forceps for separating snail tissues to detect the presence of sporocysts or furcocercous cercariae characteristic of *S. japonicum*.

### Study area

**Fig. 1:**
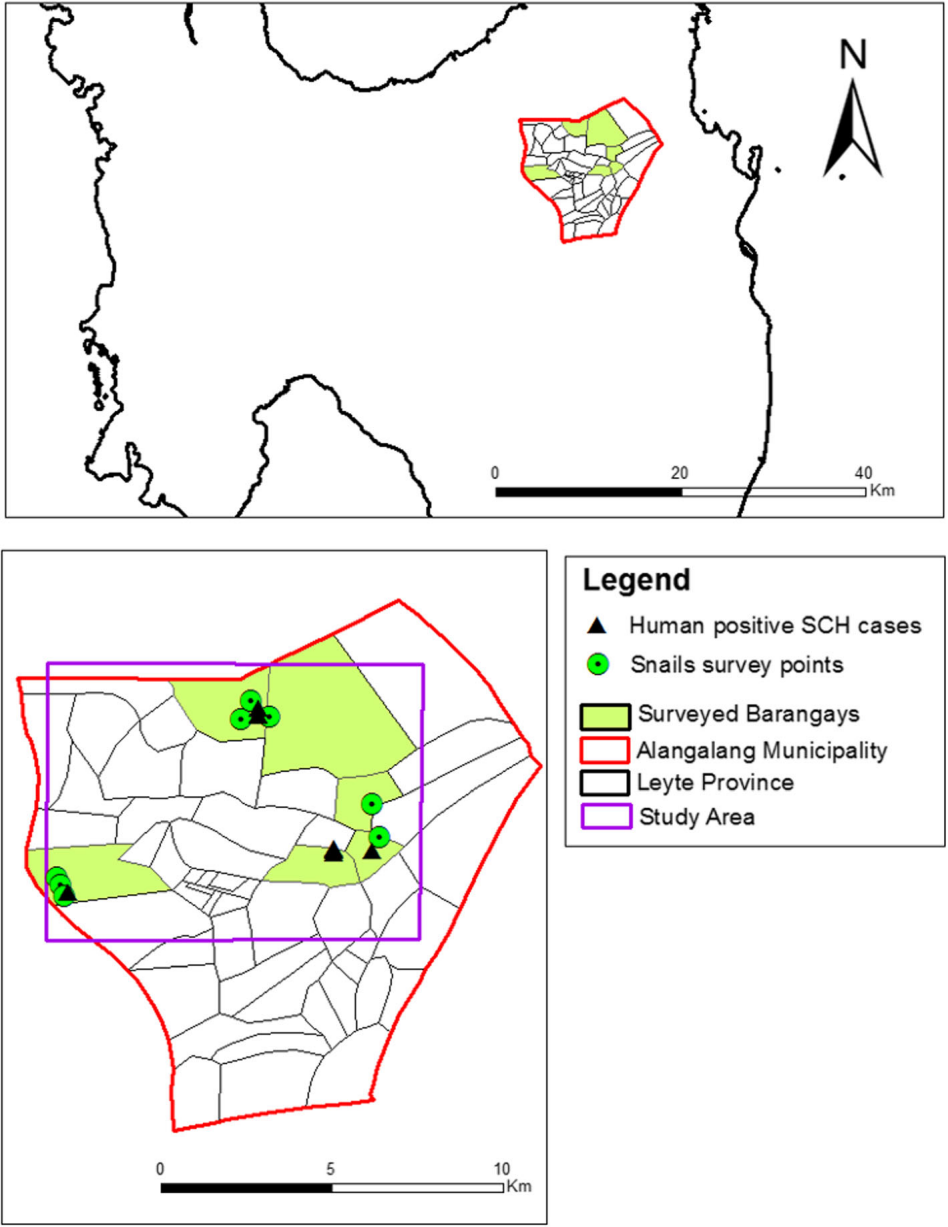
Selected study area

For the purpose of this study, it was decided to work at a local spatial scale in the Province of Leyte, due to the localized nature of the surveys and the high endemicity of the disease [28]. For the analysis, we identified a small area surrounding surveyed points (Fig. 1). This was done in order to select only surveyed barangays and to include information of all risk factors, avoiding areas without survey information (Fig. 1).

### Environmental and geographical data

Exposure risk factors of SCH transmission are associated with the environment (i.e. moisture, temperature, rainfall and water characteristics), the topography (i.e. elevation, slope) of the area [2, 10, 20, 21, 29] and snail infection status [23, 24, 30, 31]. In the endemic provinces of the Philippines, exposure to snails is mostly driven by the local topography, land use and the physical and chemical components of the water and soil [32]. We included elevation, slope, land use, nearest distance to water bodies and snail infection rates as exposure risk factors. Elevation was obtained as a raster file from Aster GDEM version 2 from USGS [33]. Vector layers for land use, river and road network were obtained from the OpenStreetMap (OSM) project [34]. OSM land use and land cover products use information from GlobeLand30 (GL30), which is a new generation of 30 m land cover maps [35–37]. The OSM road and river networks are incomplete and contain errors in their connectivity. To account for this, we edited roads and rivers, and digitalized footpaths using Google Earth images. The vector layer for snail infection rate was obtained from the recorded surveys (Table 1). Slope was derived from elevation by using the Terrain Analysis tool from Quantum GIS version 2.6 [38].

**Table 1:** Categorization of exposure risk factors

Risk factor (weight)	Spatial resolution	Temporal resolution	Data type	Coordinate system	Data source	Hypothetical link	Classification	*π* weights	Based upon
Elevation (0.03)	~ 30 m at equator	na	Raster	EPSG:4326	Aster GDEM V2 from USGS	While elevation decreases, the risk of infection increases	High risk: < 900 m	0.70	[32, 51, 56]
							Medium risk: 900–2300 m	0.25	
							Low risk: > 2300 m	0.05	
Land use (0.26)	~ 30 m	2-3-2017	Vector	EPSG:4326	OpenStreetMap project	Wet surfaces are more suitable to ahigher risk of infection	Very high risk: wet soils	0.42	[32, 57]
							High risk: water bodies	0.29	
							High and medium risk: Agriculture land and grass	0.16	
							Medium and low risk: forest and natural areas	0.08	
							Low risk: barren land	0.02	
							Very low risk: built land	0.03	
Slope (0.13)	~ 30 m at equator	na	Raster	EPSG:4326	Derived from elevation	At more flat surfaces the risk of infection increases	High risk: < 11 degrees	0.70	[49, 51]
							Medium risk: 11–30 degrees	0.23	
							Low risk: > 30 degrees	0.07	
Distance to water bodies (0.50)	30 m	2-3-2017	Raster	EPSG:32651	Derived from roads, urban areas, river network and water bodies from the OpenStreetMap project	While distance to water bodies decreases, the risk of infection increases	High risk: < 1000 m	0.74	[51, 52, 58]
							Medium risk: 1000–5000 m	0.21	
							Low risk: > 5000 m	0.05	
Snail infection rate (0.06)	na	2015–2016	Vector	EPSG:4326	Derived from recorded surveys	While snail infection rate increases, the risk of infection increases	High risk: > 3.6%	0.65	[23, 24, 30, 31]
							Medium risk: 0.5–3.6%	0.28	
							Low risk: < 0.5%	0.07	

*Abbreviation*: na, not applicable

Distance to water bodies was calculated using the closest facility network analysis tool from ArcGIS version 10 [39]. Firstly, we corrected for topology errors such as duplicate lines, presence of dangles and multipart geometries in the river and road network. Secondly, communities were loaded as incidents (261 points), and contact river points as facilities (42 points). Thirdly, we used the closest facility tool to find the nearest river from an urban area following a road. Finally, we interpolated the distance to the nearest water source using ordinary kriging from the *gstat* package in R [40] and saved the map as a raster file.

### Snail infection rate map

We constructed a trend surface that represents snail infection rate for the whole study area, thus using data of all the points to predict at unknown locations (i.e. global interpolation). It fits a mathematically defined surface through the data points (i.e. deterministic interpolation) to discover smoother (i.e. inexact interpolation) regional and local trends. It is similar to a three dimensional regression surface obtained with linear regression, where coordinates *s*_*i*_ = (*x*_*i*_, *y*_*i*_) are used as predictors. The interpolated value *z*(*S*_*i*_) for a first and second order polynomial is represented in equations 1 and 2, respectively. *z*(*S*_*i*_) represents infection rate values (number of positive cases/number of sampled snails) at location *i*.

1 $$ {z}^{\ast}\left({s}_i\right)={\beta}_0+{\beta}_1{x}_i+{\beta}_2{y}_i $$

2 $$ {z}^{\ast}\left({s}_i\right)={\beta}_0+{\beta}_1{x}_i+{\beta}_2{y}_i+{\beta}_3{x}_i^2+{\beta}_4{y}_i^2+{\beta}_5{x}_i{y}_i $$

**Fig. 2:**
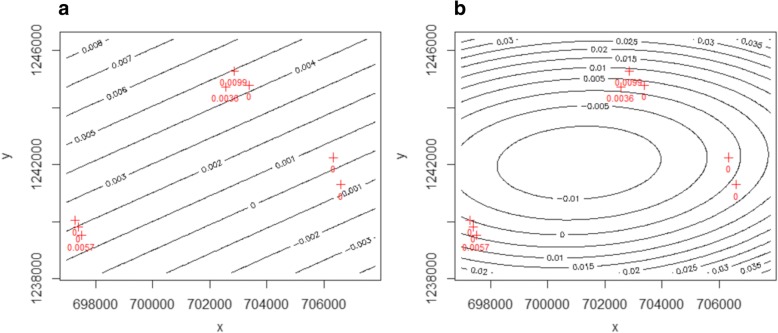
First order (**a**) and second order (**b**) polynomial trend surface. Red crosses represent the original surveyed snail infection locations

Figure 2a, b shows the resulting surfaces for the first and second order polynomials, respectively. Figure 2a shows low risk probability values (Table 1), from -0.003 to 0.008. These values do not match the original surveyed values. Figure 2b shows low and medium risk probability values from -0.01 to 0.035. These values show a better fit to the original surveyed values showed in red.

**Fig. 3:**
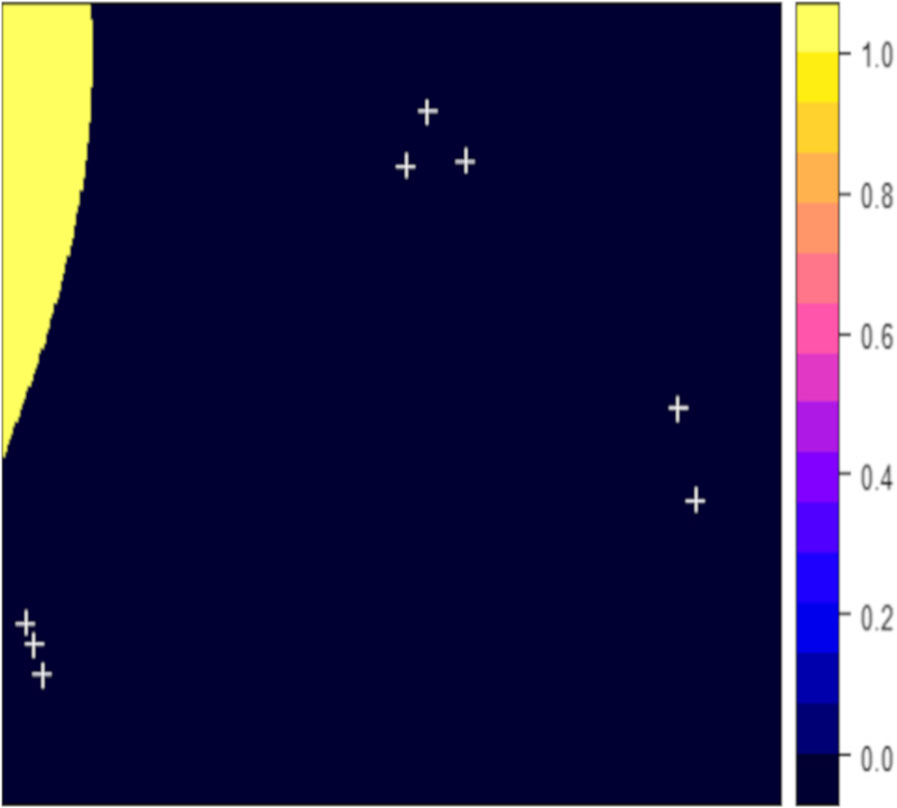
Predicted probability of snail infection values using generalized linear regression model. Colour scale represent probability values from 0 to 1. Snail survey locations are represented by white crosses

To remove the occurring negative values, we fitted a multiple linear regression by applying a generalized linear regression model using equation 2. In this case *z*(*S*_*i*_) was the infection status for each location *i*, 1 indicates an infected case and 0 a non-infected case. The resulting prediction from Fig. 3 shows only positive predicted values but very large standard errors (28.7 to 3e+13). Besides, none of the predictions approximate the original surveyed values. Finally, the second order trend surface (Fig. 2b) map was used for the analysis since it better fitted the original surveyed values.

### Spatial Bayesian network of *Schistosoma japonicum* exposure

**Fig. 4:**
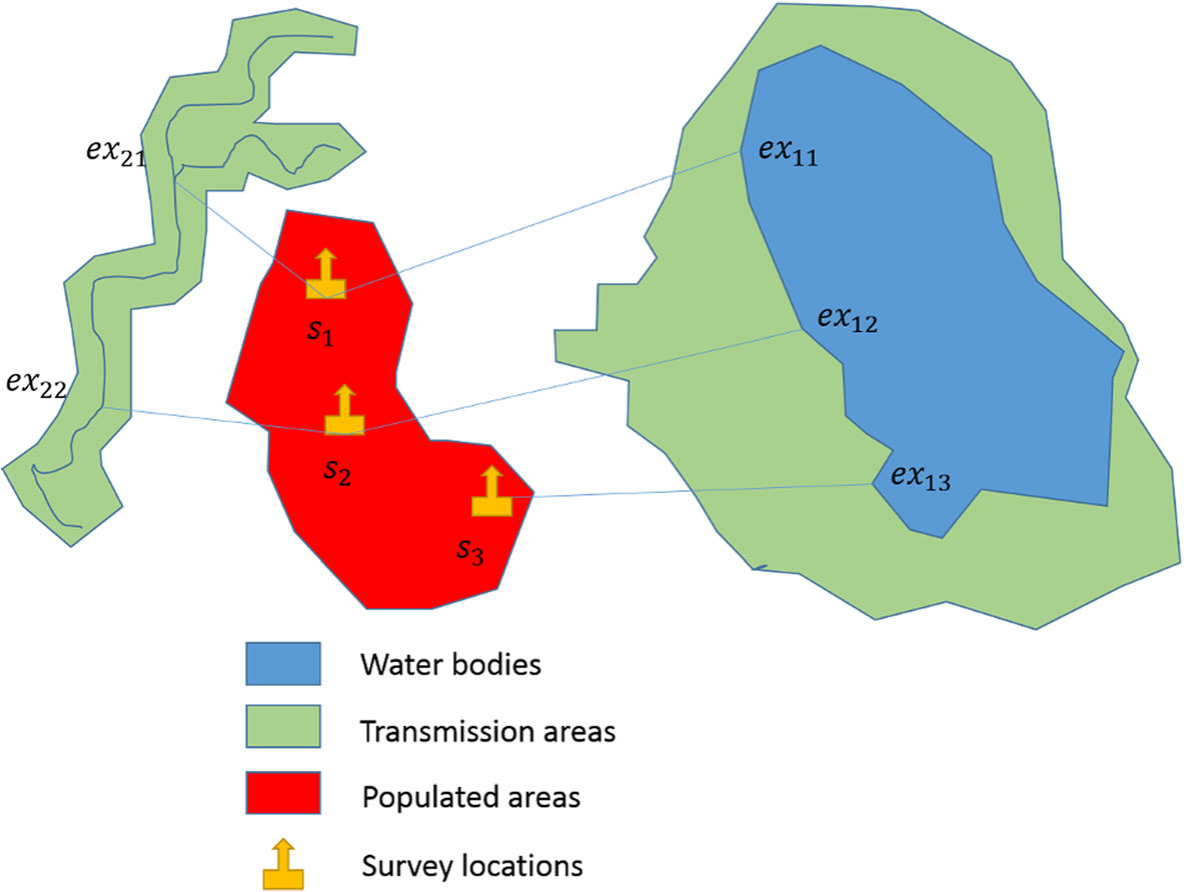
Positional mismatch in SCH modelling

We have conceptually designed a model that represents the positional mismatch between survey locations and exposure sites (Fig. 4). Locations *s*_1_and *s*_2_represent the schools, households, or other survey locations from which morbidity indicators are extracted, while *ex*_*mn*_represents the various exposure points where infections could have taken place, *m* is the corresponding number of exposure points and *n* is the corresponding survey locations related to the exposure.

Exposure areas were delineated by using spatial Bayesian networks (sBN) [41]. A Bayesian network (BN) is a probabilistic graphical model that captures the various conditional dependencies of a set of random variables (discrete or continuous) [42, 43], into a joint probability distribution by means of a directed acyclic graph (DAG) [44, 45]. A BN for a set of random variables *X* is defined by the pair (*D*, *P*). Here, *D* is the DAG and *P* is the set of probability distributions for all variables in the network. Each variable *x* with parents *pa*(*x*) has a conditional probability *p*(*x*| *pa*(*x*)). For a BN with a set of discrete (*I*) variables, the joint probability distribution factorizes into equation 3 [42]. This is the joint probability distribution as the product of all conditional probabilities specified in a BN:

3 $$ p(X)={\prod}_{i=1}^Ip\left({x}_i| pa\left({x}_i\right)\right) $$

**Fig. 5:**
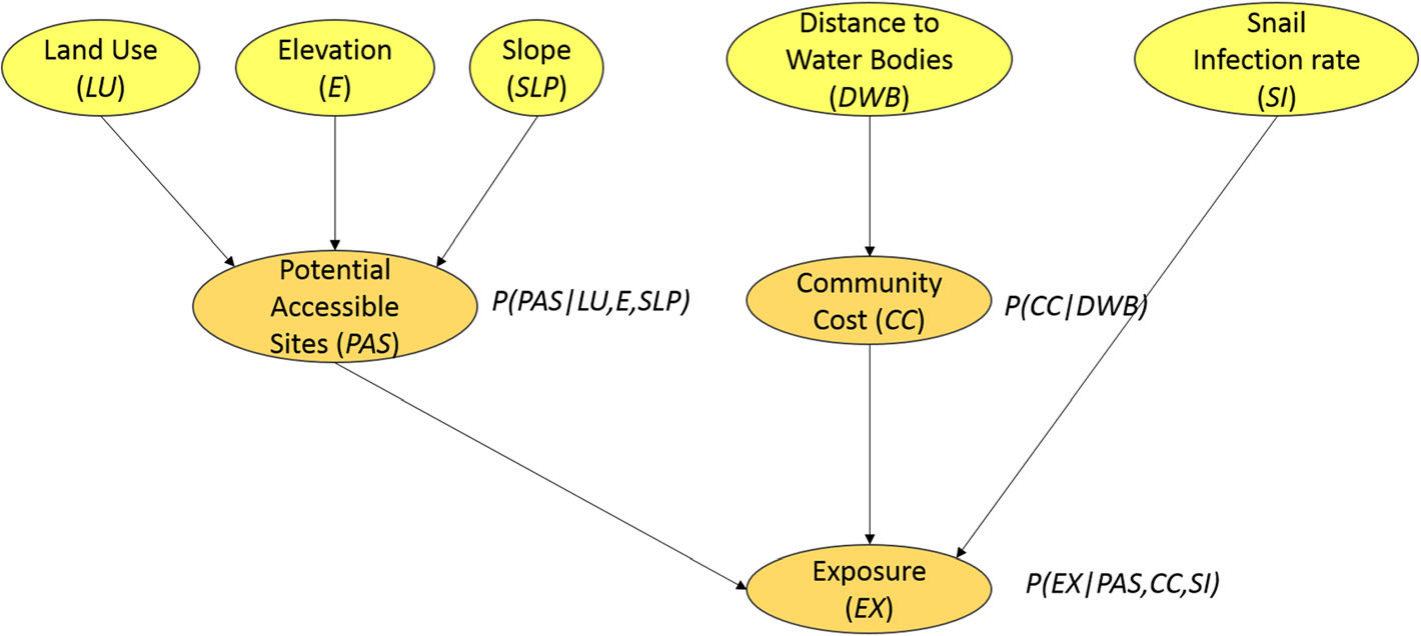
Spatial Bayesian network for SCH exposure. Yellow and orange nodes are observable and latent risk factors, respectively

The schistosomiasis exposure sBN defines exposure areas in a probabilistic way, by allowing the combination of various probability distributions from a set of random spatial variables [44]. We have constructed a DAG for exposure areas (Fig. 5), where each random variable is represented as a node. Nodes are connected by directed links or edges that express probabilistic relationships between the variables [43]. Three types of random variables can be found including (i) an observable discrete variable [land use (*LU*)]; (ii) observable continuous variables [elevation (*E*), slope (*SLP*), distance to water bodies (*DWB*) and snail infection rates (*SI*)]; and (iii) latent discrete variables [potential accessible sites for snails (*PAS*), community cost (*CC*) and exposure (*EX*)]. The direction given in the link between variables, for instance from *LU* to *PAS*, encodes a direct causal dependence of *PAS* on *LU*; the node *LU* is known then as the parent of *PAS* [45].

All continuous variables (E, SLP, DWB and SI) were discretized into different categories, given that high or low levels of exposure could occur at various ranges of risk factor values. We established hypothetical relationships between the risk factors and the disease, and categorized the risk factors based on literature (Table 1).

Exposure is a discrete child node, which has three discrete parent nodes: *PAS*, *CC* and *SI*; its conditional probability is expressed as *p*(*EX*| *PAS*, *CC*, *SI*). *PAS* and *CC* are at the same time child nodes conditional on discrete parents. Their conditional probabilities are derived by *p*(*PAS*| *LU*, *E*, *SLP*) and *p*(*CC*| *DWB*), respectively. The joint probability distribution for our Bayesian network is given as:

4 $$ p(X)=p\left( EX| PAS, CC, SI\right).\kern0.5em p\left( PAS| LU,E, SLP\right).\kern0.5em p(LU).\kern0.5em p(E).\kern0.5em p(SLP).p\left( CC| DWB\right).\kern0.5em p(DWB).\kern0.5em p(SI) $$

Equation 4 encodes assumptions of this research about direct dependencies between variables and indicates which node probability tables (NPT) need to be defined [45].

#### Construction of node probability tables

After defining the structure of our sBN, a main challenge is to construct the node probability tables (NPT). NPT are probability tables associated to each child node *v* given every possible state of the set of parents of *v*. NPT are intended to capture the strength of the relationship between the node and its parents [45]. The practicality of doing this depends on the number of states of the parent and child nodes. In our sBN eight NPTs were constructed, five NPTs as prior marginal probabilities (*π*) were inserted for the set of parent nodes (*LU*, *E*, *SLP*, *DWB* and *SI*) and three NPTs as conditional probabilities linking parent and child nodes (*PAS*, *CC* and *EX*).

We inserted prior marginal probabilities for the set of discrete parent nodes as weights. Weights were calculated using the eigen vector derived from a pairwise comparison matrix using Saaty’s comparison table [46]. Saaty [46] uses a scale of numbers (i.e. scale of judgement) to indicate how many times a factor is more dominant than another with respect to a criterion used for their comparison. In this case, the criterion is the risk of infection assigned to each parent node category given by literature (Table 1). Consistency indexes and ratios were calculated in order to measure the consistency of the judgements. Consistency ratios lower than 10%, indicate that our judgements are acceptable, while consistency ratios higher than 10% indicate untrustworthy judgements or random decisions. Saaty’s pairwise matrices as well as consistency indexes and ratios are included as Additional file 1: Tables S1-S7. Prior marginal probabilities for the parent nodes are shown in Table 1.

Latent variables *PAS*, *CC* and *EX* were divided into three probability categories: high, medium and low risk. Conditional probabilities for these child nodes are associated with the edges that link them to the parent nodes, and were also assigned using a pairwise comparison matrix. The criterion used to assign the scale of judgement is the strength of the hypothetical link between the risk factors and exposure. The strength of the hypothetical link was evaluated based upon three studies that evaluated the risk factors associated with schistosomiasis infection [47–49].

Hu et al*.* [47] ranked the potential importance of the schistosomiasis risk factors by means of a power detector. According to this detector, distance to water bodies is the most significant factor for disease risk, and elevation the least significant. Zhang et al*.* [48] used environmental, topographical and human behavioural factors to locate schistosomiasis active transmission sites. Their predictor capacity was compared by means of deviance analysis, used to determine the important variables to be included in a generalized additive model. As in the previous study, distance to water bodies was the most significant factor because of the smallest deviance, and elevation the least significant. Finally, Ajakaye et al*.* [49] evaluated physical and environmental risk factors to identify areas with suitable conditions for schistosomiasis transmission. They used Saaty’s comparison matrix to assign weights to each risk factor. Distance to water bodies and land use were the most significant factors, followed by elevation and slope as the least significant.

Weights obtained for each risk factor are shown in Table 1 and the conditional probabilities linking parent and child nodes are shown in Additional file 2: Tables S8-S10.

#### Deriving joint probabilities

To compute the probabilities for each category of the child nodes, *PAS*, *CC* and *EX*, conditional and marginal probabilities were used by applying equations 5, 6 and 7, respectively. Joint probability values of exposure were calculated for each polygon of analysis. In order to update the prior marginal probabilities, evidence is inserted for each spatial polygon into the observable variables (***SI***, ***LU***, ***E***, ***SLP***, ***DWB***). Bold facing indicates the insertion of evidence. Variables notation can be found in Additional file 3: Table S11.

5 $$ p\left( PAS,\boldsymbol{LU},\boldsymbol{E},\boldsymbol{SLP}\right)={\sum}_{LU,E, SLP}p\left(\boldsymbol{LU}\right)\ast p\left(\boldsymbol{E}\right)\ast p\left(\boldsymbol{SLP}\right)\ast p\left( PAS|\boldsymbol{LU},\boldsymbol{E},\boldsymbol{SLP}\right) $$

6 $$ p\left( CC,\boldsymbol{DWB}\right)={\sum}_{DWB}p\left(\boldsymbol{DWB}\right)\ast p\left( CC|\boldsymbol{DWB}\right) $$

7 $$ p\left( EX, PAS, CC,\boldsymbol{SI}\right)=\left({\sum}_{PAS, CC, SI}p\left( EX| PAS, CC,\boldsymbol{SI}\right)\ast p(SI)\left({\sum}_{LU,E, SLP}p\left(\boldsymbol{LU}\right)\ast p\left(\boldsymbol{E}\right)\ast p\left(\boldsymbol{SLP}\right)\ast p\left( PAS|\boldsymbol{LU},\boldsymbol{E},\boldsymbol{SLP}\right)\ \right)\left(\ {\sum}_{DWB}p\left(\boldsymbol{DWB}\right)\ast p\left( CC|\boldsymbol{DWB}\right)\ \right)\ \right) $$

For the implementation, polygons of analysis were constructed based on the overlaying of each risk factor (i.e. parent node). To overlay all risk factors, they were first transformed into vectors and then corrected for topology errors. Topology errors included duplicated polygons, multipart geometries and overlapping polygons.

Sensitivity analysis was used to see the relative influence of the risk factors on *PAS* and *CC*, and the relative influence of *PAS*, *CC* and *SI* on exposure. We used the sensitivity function, calculated as the degree of entropy reduction. Degree of entropy reduction *I* is the degree of change or expected difference in information bits *H* between a query variable *Q* (exposure) with *q* states and findings variable *F* (risk factors) with *f* states [50] (equation 8). A degree of entropy reduction of 0 means a query variable is independent of the varying variable.

8 $$ I=H(Q)-H(F)={\sum}_q{\sum}_f\frac{P\left(q,f\right){\log}_2\left[P\left(q,f\right)\right]}{P(q)P(f)} $$

#### Software

To work within the spatial domain we used the software Netica^TM^ 6.03 [41], which works with Bayesian networks, decision nets and influence diagrams. Evidence is inserted as cases for each polygon of analysis, and prior and conditional probabilities are inserted as tables.

#### Validation

Validation was first performed by counting all surveyed positive SCH human cases falling inside the various categories of exposure in the map. However, this introduces a positional mismatch as the surveyed positive cases were not necessarily acquired at those specific exposure points.

As a second approach for validation, we defined potential validation areas by constructing buffers around each of the positive cases. We extracted the distance to the nearest water body for each surveyed point using the distance map previously generated. Extracted distance values were used as distance buffers generated around positive cases. Buffers completely containing other buffers were grouped. We counted the number of positive cases falling inside each group and calculated the mean probability of exposure within the grouped buffers.

## Results

### Exposure network

**Fig. 6:**
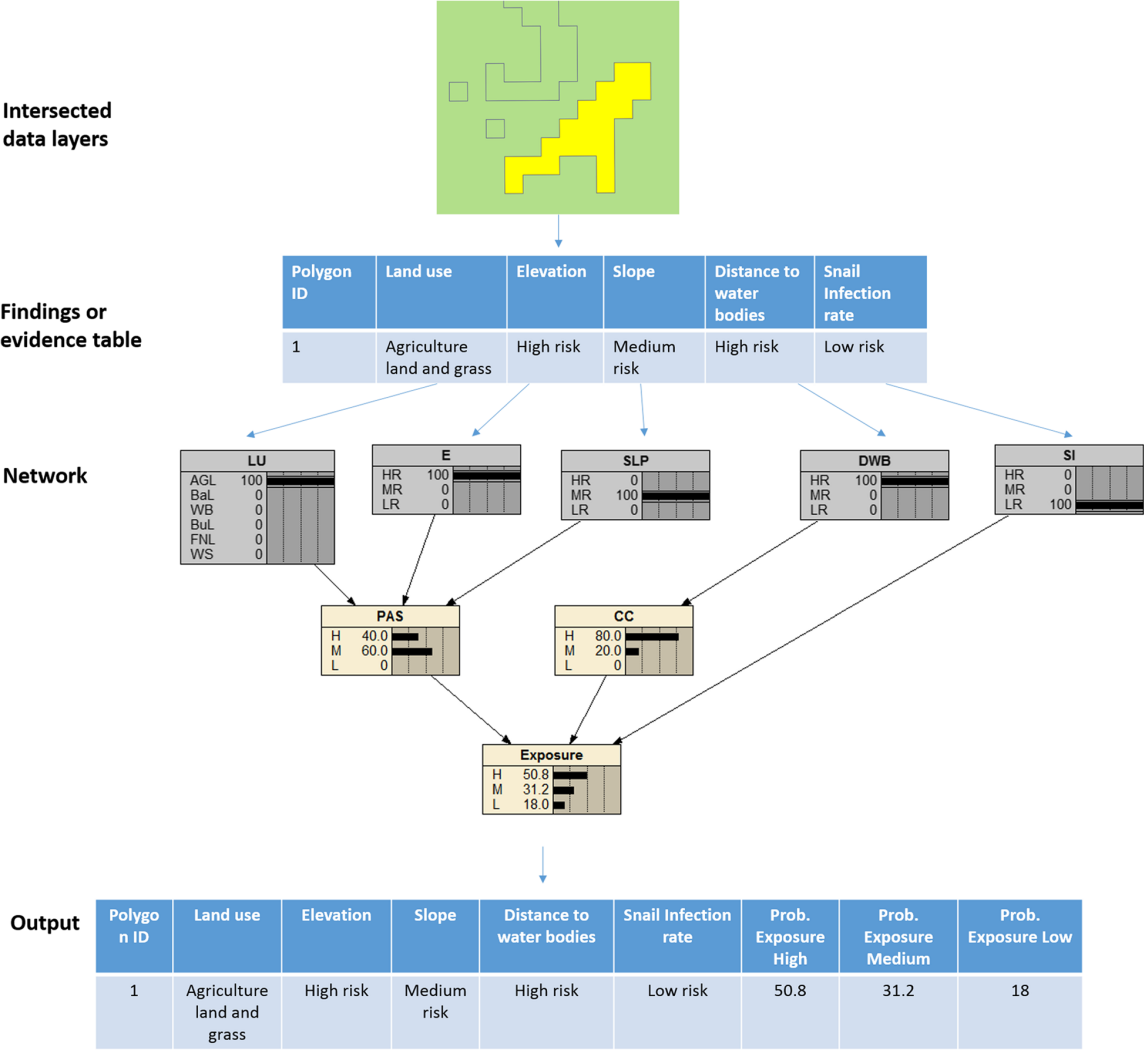
Probabilities of exposure in the Bayesian network

High (> 50%), medium (35–50%), low (20–35%) and very low (< 20%) probabilities of exposure were derived from the proposed exposure network. This is exemplified in Fig. 6 for only one polygon. For this particular polygon, the probability is predominantly high (50.8%) for a high-risk elevation (< 900 m), *DWB* (< 1 km), and *LU* (agriculture land and grass), a medium risk slope (11–30°), and a low risk *SI* (< 0.5%).

**Fig. 7:**
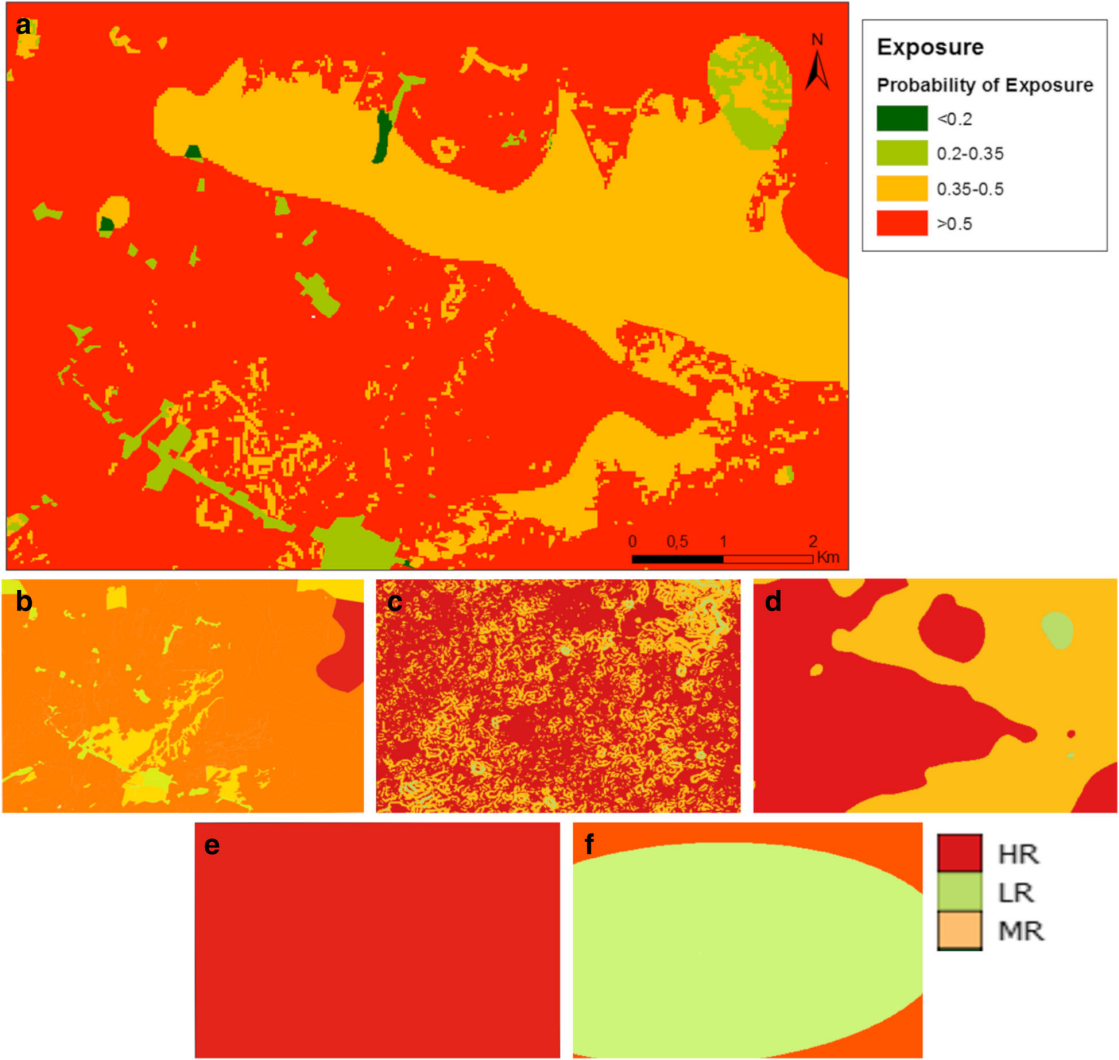
**a** Probability of exposure map. **b**-**f** Risk factors of exposure: land use (**b**); slope (**c**); distance to water bodies (**d**); elevation (**e**); snail infection rates (**f**)

Very low probability values of exposure (< 20%) were found in built-up areas, medium risk *DWB* (1–5 km), slopes < 30° and low and medium (0.5–3.6%) risk of snail infection, but also in agriculture and grass land with *DWB* > 5 km and slopes > 30° (Fig. 7). Low probabilities of exposure (20–35%) were found in built-up areas with slopes < 30°, low risk of snail infection, and within *DWB* < 1 km, but also in agriculture and grass land in *DWB* > 5 km. Medium probability values (35–50%) were found in agriculture and grass land and forest areas, in slopes > 11°, low risk of snail infection, and *DWB* < 1 km, but also in slopes < 30°, medium risk of snail infection and *DWB* from 1 to 5 km. High probability of exposure values (> 50%) were found in wet soils with slopes < 30°, with *DWB* from 1 to 5 km and medium risk of snail infection, but also in agriculture and grass land with *DWB* < 1 km and low risk of snail infection.

Based on the degree of entropy reduction, our sensitivity results show that the risk factor with the highest degree of change is *PAS* followed by *SI* and *CC.* Within PAS, land use has the highest degree of change and elevation has the lowest, showing that the most influential risk factors on exposure are land use, snail infection rate and distance to water bodies in that order, and the least influential factors are slope and elevation (Table 2).

**Table 2:** Sensitivity of exposure to risk factors using entropy reduction (variables are listed in order of influence on exposure)

Node	Degree of entropy reduction	% of influence to the network
*PAS*	0.07149	28.0
*SI*	0.06524	25.3
*CC*	0.04708	18.3
*LU*	0.04138	16.0
*DWB*	0.02868	11.1
*SLP*	0.00291	1.1
*E*	0.00066	0.2

**Fig. 8:**
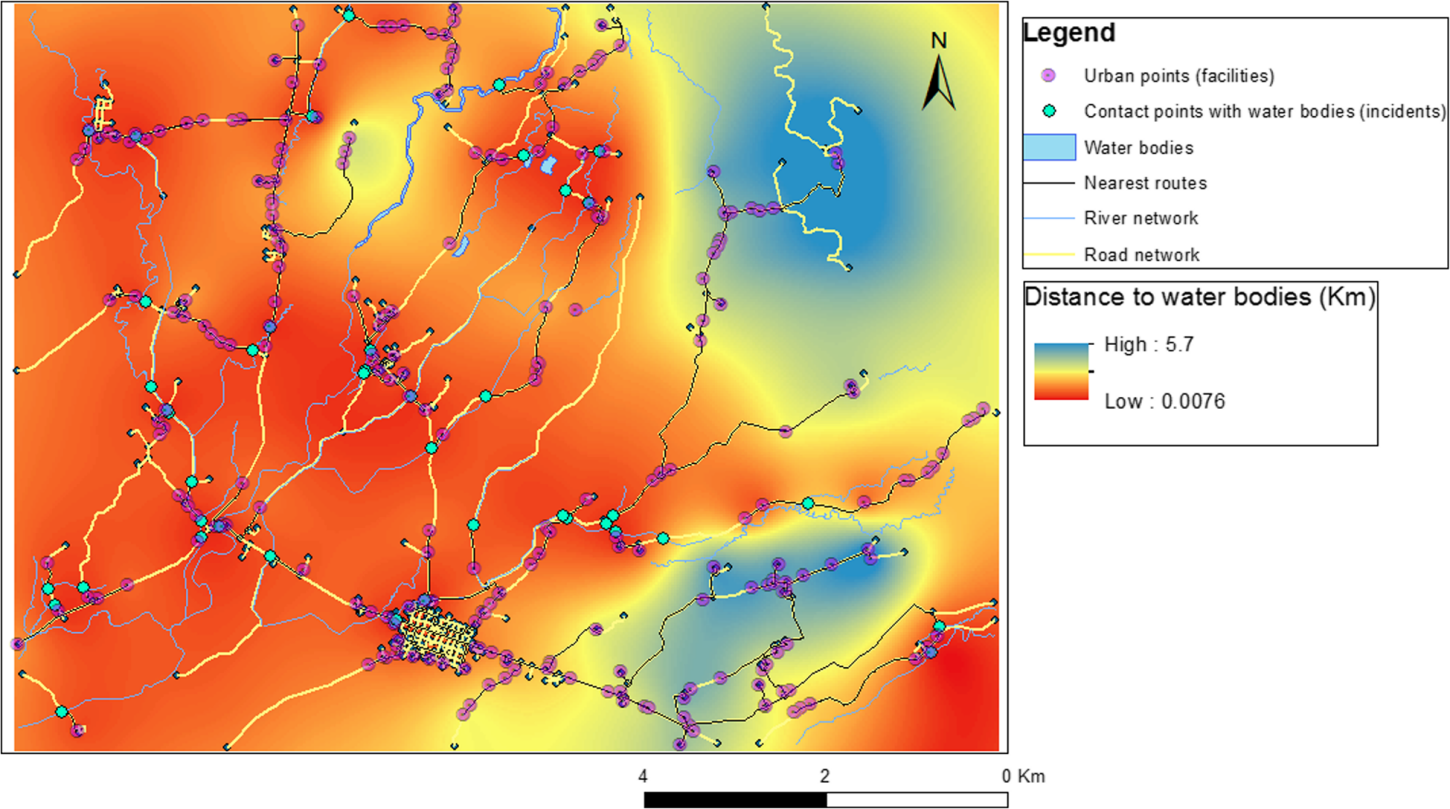
Nearest route calculation from urban points to water bodies and DWB ordinary kriging interpolation

Our findings show that approximately 63% of the study area has high probability of exposure values (> 50%). This is mainly explained by the predominance of agricultural fields in the area (Fig. 7b) and the distance to water bodies results, which indicate that approximately 80% of the urban areas can access water bodies following routes < 500 m. Lowest and highest distance values between urban areas and water bodies are 7.6 m and 5.7 km, respectively, with a mean of 1.4 km (Fig. 8).

### Validation

**Fig. 9:**
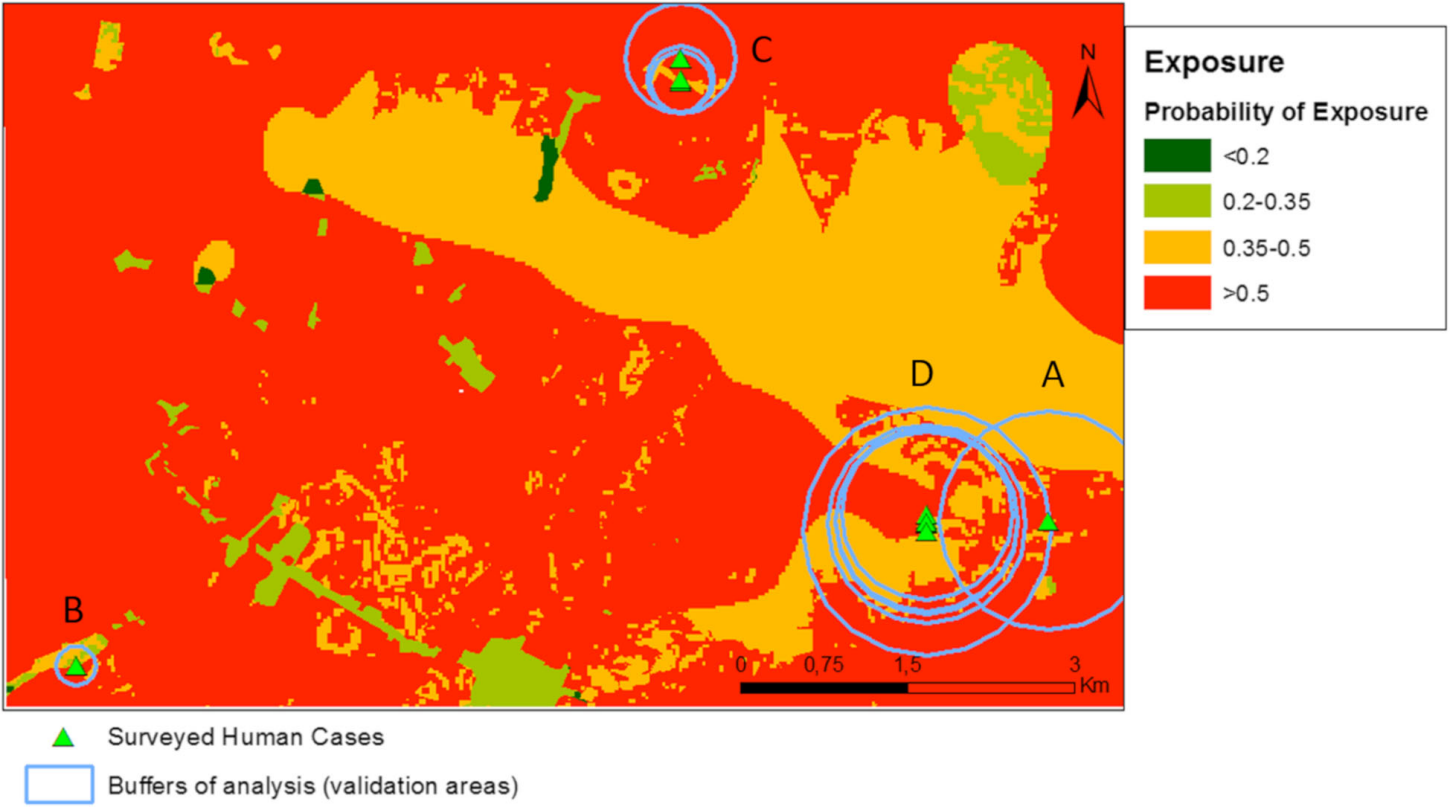
Buffers around surveyed human cases points. Letters show the grouped buffers based on points location

**Fig. 10:**
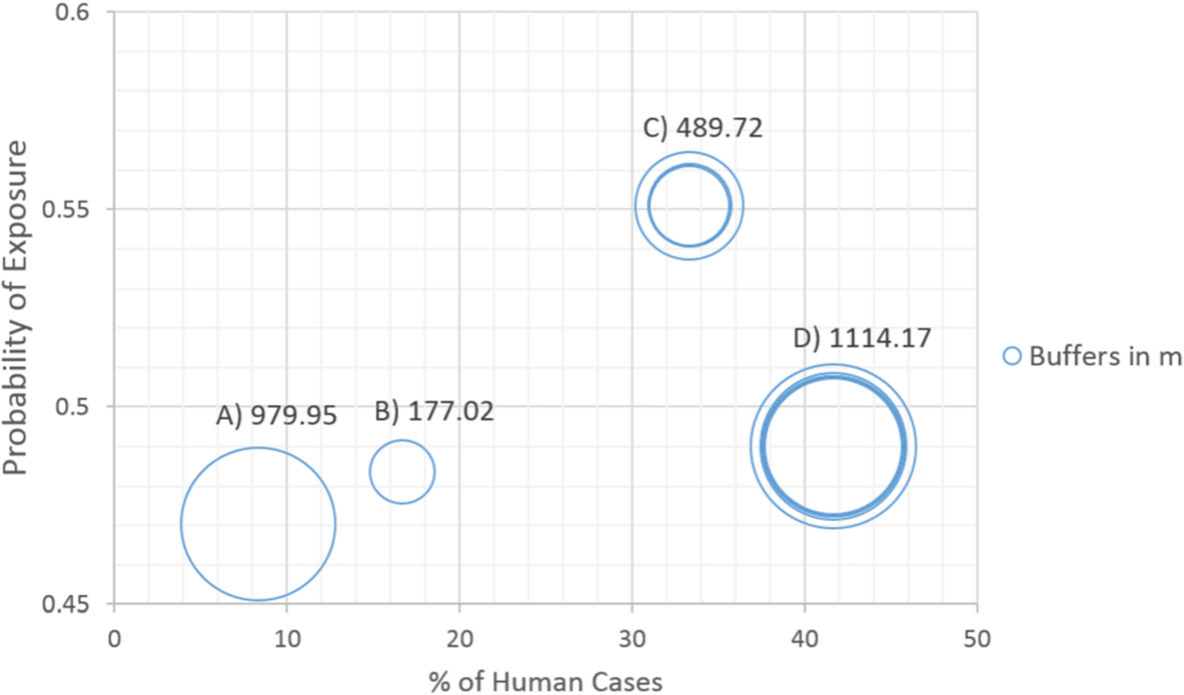
Probability of exposure vs percentage of human cases. Labels correspond to the grouped buffers visualized in Fig. 9

**Fig. 11:**
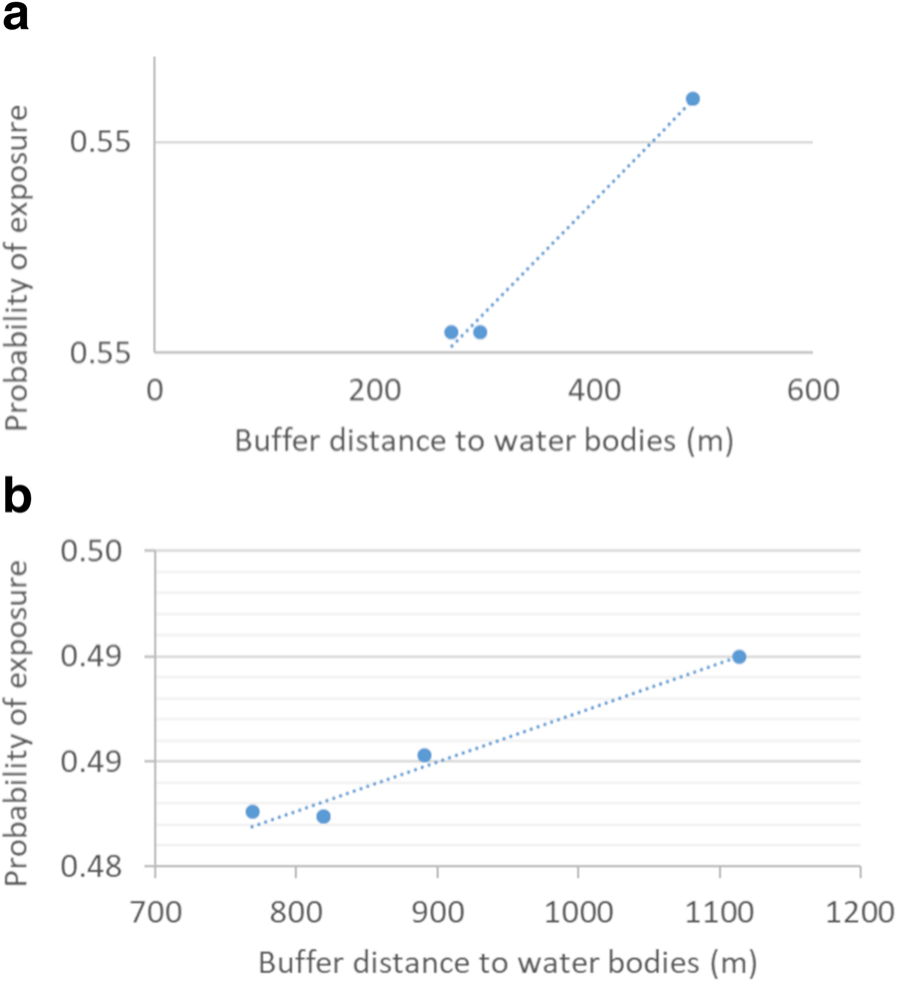
Distance to water bodies versus probability of exposure. Plotted values for **a** Group C and **b** Group D

For the first validation, the results show an increase in the probability of exposure as the proportion of human cases also increases, except for 17% of human cases where a reduction in the probability of exposure of 35.8% can be observed (Table 3). For the second validation, four groups of buffers were observed: Group A with one positive case, Group B with two positive cases and Groups C and D with four and five positive cases, respectively (Fig. 9). A low correlation was found between probability of exposure and percentage of human cases within the groups (linear correlation, *R*^2^ = 0.3). For the first three groups (A, B and C) the probability of exposure increases while the percentage of human cases also increases. For Group D, the group with more positive cases, a minor decrease in the probability of exposure can be observed (Fig. 10). This could be explained by the distance to water bodies that has a negative correlation (Group C: -0.3, Group D: -0.02) with the probability of exposure values (0.47–0.55) calculated from our sBN for groups C (*R*^2^ = 0.98) and D (R^2^ = 0.96) (Fig. 11). For instance, for Groups A and B with one and two positive cases respectively, the distance to water bodies is higher for Group A (~980 m) than for Group B (~177 m), with an average exposure value of approximately 0.47 and 0.48, respectively (Fig. 10). Likewise, for Groups C and D, the distance to water bodies is higher for Group D (~1100 m) than for Group C (~490 m), with an average probability of exposure values equal to 0.55 and 0.49, respectively (Fig. 10).

**Table 3:** Percentage of human cases falling within probabilities of high exposure values

No. of human cases	% of human cases	Probability of exposure
1	8.3	41.2
2	16.7	35.8
3	25.0	50.8
6	50.0	55.6

## Discussion

Several studies have modelled snail distribution as input information for risk prediction of schistosomiasis [2, 20, 22, 47, 48, 51], in order to guide prevention (sanitary and hygiene conditions of the population) and control (mass drug administration campaigns in the community) strategies for schistosomiasis infection. These approaches are inadequate spatial decision support tools since they have not accounted for snails’ infection status or people’s exposure to infection (i.e. contact of people with snails’ sites). In this study we demonstrate a novel approach to delineate spatial areas of exposure to *S. japonicum* infection by accounting for the distribution of infected and non-infected snails, and considering the human interaction with active transmission sites. This was done by accounting for the cost of the community to access water bodies and potential sites where snails may be present.

Our results suggest that the predominance of high probabilities of exposure values (> 50%) in the study area are explained by the presence of wet soils and agriculture land in the zone, but also by the distance from urban areas to nearby water bodies (< 5 km). This was expected given that land use is a highly influencing risk factor on exposure after potential accessible sites (Table 2), and also because of the initial high weights given to *LU* and *DWB* (Table 1).

Our results demonstrate that for short distances to water bodies, the probability of a community to be exposed to *S. japonicum* is high (Fig. 8). This was explained by the probability of exposure map and the relative influence of *DWB* on exposure. Although *DWB* is the fifth influencing factor on exposure (Table 2), it is the only influencing factor on community cost, which is the third most important variable of the network (Table 2). Based on our results we propose that future studies utilise the nearest distance to water bodies following a road instead of the commonly used Euclidean distance [51–53], since the former provides a more accurate representation of community access to water bodies, as it accounts for the nearest path from human dwellings to potential infection foci.

We postulated that the proportion of human *S. japonicum* cases was higher in areas predicted to have a higher probability of exposure. Our validation procedure using overlaying proportions in the four groups of buffers surrounding nearby *S. japonicum* cases, demonstrated a positive correlation for three groups. Although the number of validation points is somewhat low for a total validation, overlying proportions of exposure to schistosomiasis infection suggest a correlation between potential areas of exposure and the disease in the presence of limited survey data.

### Utility of modelling the geographical probability of *S. japonicum* exposure

Modelled schistosomiasis exposure areas account for the transmission processes occurring between the environment containing infective stages of *S. japonicum* or intermediary hosts (snails), and the susceptible hosts (humans and livestock). From a public health perspective, the provision of maps that define the geographical limits of probability of exposure to *S. japonicum* infected areas could help target local schistosomiasis control strategies to communities more likely to contact contaminated environments and thereby improve the efficiency of mass drug administration campaigns. From a spatial modelling perspective, the availability of a predictive exposure map could serve as an important base map to obtain covariate values. By relating them to indicators of disease, we could possibly account for the positional mismatch between epidemiological survey data and environmental covariates, and improve the statistical modelling of *S. japonicum* infection.

### Limitations of the study

A number of limitations should be accounted for in the interpretation of our results. Firstly, estimates of the probability of exposure are highly influenced by the availability of snail infection estimates (Table 2). Due to the localized nature of the study, it was difficult to generate an adequate surface map that could properly explain snail infection distribution, constraining this map into a binary output with low and medium risk values (Fig. 7f). This might have an impact on the results and could be further improved by an increase of the study extent, and the number of survey points. In addition, whenever these data or new knowledge becomes available, the sBN developed in this study will enable a “rapid delineation” of potential exposure areas of *S. japonicum* by facilitating a flexible integration of exposure data as risk factors, and prior information derived from literature or expert knowledge [54].

Secondly, model validation procedures could be improved by including positive and negative human cases. Collecting data on livestock infection [23, 24, 30, 31] could also serve for validation as livestock infection, particularly carabao, has been suggested to play an important role in the transmission of *S. japonicum* in the Philippines [55].

## Conclusions

In conclusion, the present study describes the nature of the positional exposure mismatch in the modelling of *S. japonicum* infection. Results of the present study suggest that the best way to address this mismatch should include the extraction of covariate values from potential exposure areas. A probabilistic method to delineate exposure areas in the absence of sufficient empirical survey data is proposed. Unlike other studies, the present sBN is adequate to delineate exposure areas based upon the contact of communities to water bodies and other potential sites of infection. We conclude that even with limited disease survey data, it is possible to define potential exposure areas for schistosomiasis. Modelled exposure areas might be used to correct for positional mismatches and significantly improve disease predictions to better guide control programs to prevent and control schistosomiasis and other water-borne infections.

## Additional files

Additional file 1: **Table S1.** Saaty’s pairwise comparison matrix for Land Use. **Table S2.** Saaty’s pairwise comparison matrix for Elevation. **Table S3.** Saaty’s pairwise comparison matrix for Slope. **Table S4.** Saaty’s pairwise comparison matrix for Distance to water bodies. **Table S5.** Saaty’s pairwise comparison matrix for Snail infection rate. **Table S6.** Saaty’s pairwise comparison matrix for all risk factors. **Table S7.** Total weights for all risk factors. Saaty’s pairwise comparison tables for the risk factors and their categories. This file also includes the calculation of consistency indexes and ratios. (XLSX 31 kb)

Additional file 2: **Table S8.** Conditional probabilities for Land Use. **Table S9.** Conditional probabilities for Community cost. **Table S10.** Conditional probabilities for Exposure. Node probability tables. Conditional probability tables used for each one of the latent variable nodes: exposure (EX), potential accessible sites (PAS) and community cost (CC). (XLSX 16 kb)

Additional file 3: **Table S11.** Abbreviations used in the manuscript and variable notations. (XLSX 14 kb)

## Abbreviations

BN: Bayesian network
DAG: Directed acyclic graph
GIS: Geographical information systems
GPS: Global positioning systems
NPT: Node probability tables
sBN: Spatial Bayesian network
SCH: Schistosomiasis
